# Human settlement of East Polynesia earlier, incremental, and coincident with prolonged South Pacific drought

**DOI:** 10.1073/pnas.1920975117

**Published:** 2020-04-06

**Authors:** David A. Sear, Melinda S. Allen, Jonathan D. Hassall, Ashley E. Maloney, Peter G. Langdon, Alex E. Morrison, Andrew C. G. Henderson, Helen Mackay, Ian W. Croudace, Charlotte Clarke, Julian P. Sachs, Georgiana Macdonald, Richard C. Chiverrell, Melanie J. Leng, L. M. Cisneros-Dozal, Thierry Fonville

**Affiliations:** ^a^School of Geography and Environmental Science, University of Southampton, Highfield SO17 1BJ Southampton, United Kingdom;; ^b^Anthropology, School of Social Sciences, University of Auckland, 1142 Auckland, New Zealand;; ^c^School of Oceanography, College of the Environment, University of Washington, Seattle, WA 98195;; ^d^International Archaeology LLC, Honolulu, HI 96826;; ^e^School of Geography, Politics and Sociology, Newcastle University, NE1 7RU Newcastle upon Tyne, United Kingdom;; ^f^Geosciences Advisory Unit (GAU)-Radioanalytical, National Oceanography Centre, University of Southampton, SO14 3ZH Southampton, United Kingdom;; ^g^Department of Geography and Planning, School of Environmental Sciences, Liverpool University, L69 7ZT Liverpool, United Kingdom;; ^h^National Environmental Isotope Facility, British Geological Survey, NG12 5GG Nottingham, United Kingdom;; ^i^Centre for Environmental Geochemistry, School of Biosciences, University of Nottingham, LE12 5RD Loughborough, United Kingdom;; ^j^National Environmental Isotope Facility, Radiocarbon Laboratory, Scottish Universities Environmental Research Centre, University of Glasgow, G75 0QF East Kilbride, United Kingdom

**Keywords:** Polynesian voyaging, East Polynesian colonization, biomarkers, drought, palaeoclimate

## Abstract

The timing of human colonization of East Polynesia, a vast area lying between Hawai‘i, Rapa Nui, and New Zealand, is much debated and the underlying causes of this great migration have been enigmatic. Our study generates evidence for human dispersal into eastern Polynesia from islands to the west from around AD 900 and contemporaneous paleoclimate data from the likely source region. Lake cores from Atiu, Southern Cook Islands (SCIs) register evidence of pig and/or human occupation on a virgin landscape at this time, followed by changes in lake carbon around AD 1000 and significant anthropogenic disturbance from c. AD 1100. The broader paleoclimate context of these early voyages of exploration are derived from the Atiu lake core and complemented by additional lake cores from Samoa (directly west) and Vanuatu (southwest) and published hydroclimate proxies from the Society Islands (northeast) and Kiribati (north). Algal lipid and leaf wax biomarkers allow for comparisons of changing hydroclimate conditions across the region before, during, and after human arrival in the SCIs. The evidence indicates a prolonged drought in the likely western source region for these colonists, lasting c. 200 to 400 y, contemporaneous with the phasing of human dispersal into the Pacific. We propose that drying climate, coupled with documented social pressures and societal developments, instigated initial eastward exploration, resulting in SCI landfall(s) and return voyaging, with colonization a century or two later. This incremental settlement process likely involved the accumulation of critical maritime knowledge over several generations.

## Significance

We combine indicators from lake sediments with archaeological records that identify an earlier and incremental arrival of humans in East Polynesia than indicated by current models. We use lake sediments to reconstruct a quantitative, multiproxy hydroclimate sequences from Vanuatu, Samoa, and the Southern Cook Islands and combine these with published data to show that the timing of human migration into East Polynesia coincided with a prolonged drought. We postulate this regional drought was a significant contributory factor in eastward exploration and subsequent colonization of the Southern Cook Islands and beyond. The return of wetter conditions in East Polynesia after c. AD 1150 supported subsequent colonization of other central islands and, eventually, migration into far eastern and South Polynesia.

Colonization of the vast eastern Pacific, with its few and far-flung archipelagos, was a remarkable achievement in human history. Yet the timing, character, and drivers of this accomplishment remain poorly understood. Specifically, the final phase of human dispersal occurred nearly 2 millennia after colonization of West Polynesia (Tonga and Samoa), raising questions about why voyaging was apparently discontinuous and why it resumed after such a prolonged standstill.

Recent chronometric studies, particularly those using short-lived materials (SLMs; ≤10 y) with little in-built age (1, 2), along with U-Th dating (3), have gone some way toward resolving the chronology of human settlement in East Polynesia, a region distinguished by a high degree of cultural, biological, and linguistic similarity (4). High-precision chronologies are now available for several central archipelagos, including the Cook (3, 5), Society (6), and Marquesas islands (7), along with Mangareva (8), unambiguously placing Polynesians in the central East Polynesian core by the 12th century AD.

Less attention has been directed to the drivers of East Polynesian exploration and eventual settlement. Some stress the importance of demographic processes and resource deterioration (anthropogenic or otherwise) (9) as “push” factors, but evidence from West Polynesian archaeological sites has been equivocal. Others have shown that wind patterns and the 800- to 1,200-km water gap between Samoa–Tonga and the Southern Cook Islands (SCIs) constituted a critical navigational threshold (10). This in combination with greater interarchipelago distances, smaller island sizes, and arcs of landfall shaped by novel wind conditions may have required watercraft improvements and new voyaging strategies that took time to develop (10). Pulses of Pacific colonization may also be linked to intensified periods of the El Niño Southern Oscillation (ENSO) (11). Problematically, assessing the influence of climate on Polynesian migrations has been hampered by the short length (<600 y) of Pacific paleoclimate proxy records and/or their absence from the areas that lie at critical migration thresholds.

**Fig. 1.**
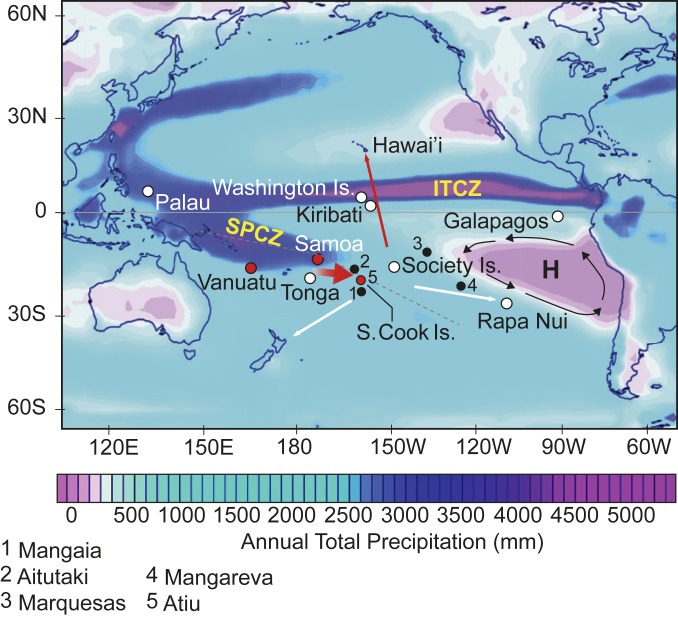
Pacific region showing average annual precipitation patterns (GPCP satellite-gauge 1979 to 2018), Intertropical Convergence Zone, South Pacific Convergence Zone, and southeast Pacific High (H) in relation to regional geography. Dashed line denotes main axis of the SPCZ. Sites with lake sediment proxy records (this study) are shown as red circles; other proxy records used herein are shown as white circles. Red arrows show the initial migration east into the gateway islands—Southern Cook Islands and Society Islands, and north to the Marquesas and Hawai‘i, the latter dated to c. 1000 to 1200 AD. White arrows show subsequent migrations to Polynesian margins c. 1150 to 1300 AD (see text for details).

We report multiproxy analyses from lake sediment archives aimed at understanding the paleoclimatic context and timing of the earliest human excursions into the East Polynesian realm. The timescales for our sediment records were produced by Bayesian age–depth modeling of radiometric ages (^210^Pb and accelerator mass spectrometry [AMS] ^14^C), with greater weight attributed to ^14^C ages obtained for SLMs, mainly terrestrial plant leaf macrofossils. Our records derive from three archipelagos that comprise a west-to-east transect along the southern boundary of the South Pacific Convergence Zone (SPCZ), a major Pacific Ocean feature (Fig. 1): Vanuatu (Efate), Samoa (‘Upolu), and the SCIs (Atiu). Vanuatu is particularly sensitive to movement of the SPCZ (12), while Samoa is identified by voyaging simulations (13), material culture, and biological evidence (4) as a likely departure point for East Polynesian explorers. Temporally relevant paleoclimate records from these two archipelagos offer insights into conditions surrounding eastward Polynesian expansion. The Southern Cook Islands, the most proximate archipelago to the potential West Polynesian homeland, have long been recognized as a likely gateway archipelago for East Polynesian colonists (4, 10, 14). More generally, the considerable time depth of our three sediment archives (Vanuatu: c. 1,750 y; Samoa: c. 10,600 y; Cook Islands: c. 6,550 y) inform on conditions before, during, and after human dispersal into the cultural region of East Polynesia.

## Study Sites

Lake Emoatul (17°43′57.48″S, 168°24′53.58″E), Efate Island, Vanuatu is a closed, 0.3-km^2^ freshwater body at an altitude of 119 metres above sea level (m.a.s.l.) within a relatively small catchment (0.98 km^2^) surrounded by moist montane forest with limited subsistence gardening and arboriculture. The lake is situated on a Pleistocene raised coral reef dated to c. 120,000 B.P. The lake has a maximum depth of 7.1 m and is meromictic with a chemocline at 6 m resulting in a suboxic (dissolved oxygen 5.0% saturation; sat) hypolimnion. Our 3.4-m sediment core was characterized by laminated gyttja throughout. Final Bayesian age–depth model uncertainties around the potential period of initial East Polynesian colonization (c. 900 to 1200) were ±85 y (2σ) (*SI Appendix*, Fig. S2 and Table S1). The resultant age–depth curve indicates sediment accumulation averages of 6 y⋅cm^−1^.

Lake Lanoto‘o (13°54′37.73″S, 171°49′39.72″W), ‘Upolu Island, Samoa is a closed freshwater body within an extinct volcanic crater at 760 m.a.s.l. The crater dates to around 1 to 0.1 million y old and has deeply weathered, silty-loam, red lateritic soils of 2 to 6% organic carbon content (15, 16). The freshwater lake is 0.11 km^2^ in area with a maximum depth of 17 m and a closed catchment area of 0.23 km^2^. The lake is surrounded by moist montane forest with a relatively undisturbed cover of *Dysoxylum huntii* (17). Lake Lanoto‘o is meromictic with a chemocline at a 10-m depth resulting in a suboxic (dissolved oxygen 10% sat) hypolimnion. The sediment sequence is gyttja throughout, with some laminations—the main color variations resulting from terrigenous in-wash (18). The resulting Bayesian age–depth model provides 2σ uncertainties of ±59 y around the period of potential migration into East Polynesia (c. AD 900 to 1200) (*SI Appendix*, Fig. S2 and Table S1). The resultant age–depth curve indicates sediment accumulation averages of 30 y⋅cm^−1^.

Lake Te Roto (20°0′34.99″S, 158°7′24.83″W), Atiu Island, Southern Cook Islands is a 26.9-km^2^ island composed of a highly weathered volcanic core and an encircling raised limestone rim or makatea of 3- to 6-m height on the seaside (17). The small (0.03-km^2^) brackish lake (surface water salinity 6 ppt [parts per thousand]; bottom salinity 16 ppt) of Te Roto lies at an elevation of 0.9 m.a.s.l. and is <1 km from the coast. The lake is semiopen with a narrow exit tunnel through the makatea that periodically connects to the ocean (17). Some 96% of the 0.3-km^2^ lake catchment drains an eroded volcanic cone. The lake is 8.4 m deep with a chemocline at a 3-m depth, resulting in an anoxic (dissolved oxygen 10% sat) hypolimnion (18). A 7.8-m core produced a sequence consisting of laminated gyttja throughout. These individual laminae most likely represent seasonal sedimentation (18) and allow for a relative chronology of environmental change (*SI Appendix*, Fig. S1). Bayesian age–depth model uncertainties associated with the period of likely anthropogenic disturbance are ±140 y. Sediment accumulation rates are estimated at 9 y⋅cm^−1^. The sources of carbon to the lake based on bulk sediment organic C/N and δ^13^C data are dominated by soil and terrestrial vegetation (18). The associated age–depth model is constrained by eight AMS ^14^C results across the depth interval of 263 to 182 cm, but there is scatter arising from measurements for bulk organic materials (*SI Appendix*, Fig. S2). BACON, the R software used for Bayesian age–depth modeling, reduces the effects of outlying dates because the ages are modeled using a Student’s *t* distribution with wide tails (19). Given the greater confidence generated by ^14^C dating SLMs, these ages were assigned more narrow Gaussian error distributions reflecting their greater reliability.

Previous coring at Lake Te Roto suggested initial human activities in the catchment as early as c. AD 590 to 765 (2σ) (17), but this single early date has been controversial. No early settlement sites have been identified on Atiu Island but archaeological work has been limited (*SI Appendix*). The lake is inshore from the traditional Vai Piake landing, with other nearby landing sites to the north. The Lake Te Roto core most directly records human activity on Atiu but given the close interisland distances (<250 km) is likely to reflect human arrival and dispersal across the SCIs as a whole.

## Anthropogenic Signatures in Lake Te Roto, Atiu, Southern Cook Islands

**Fig. 2.**
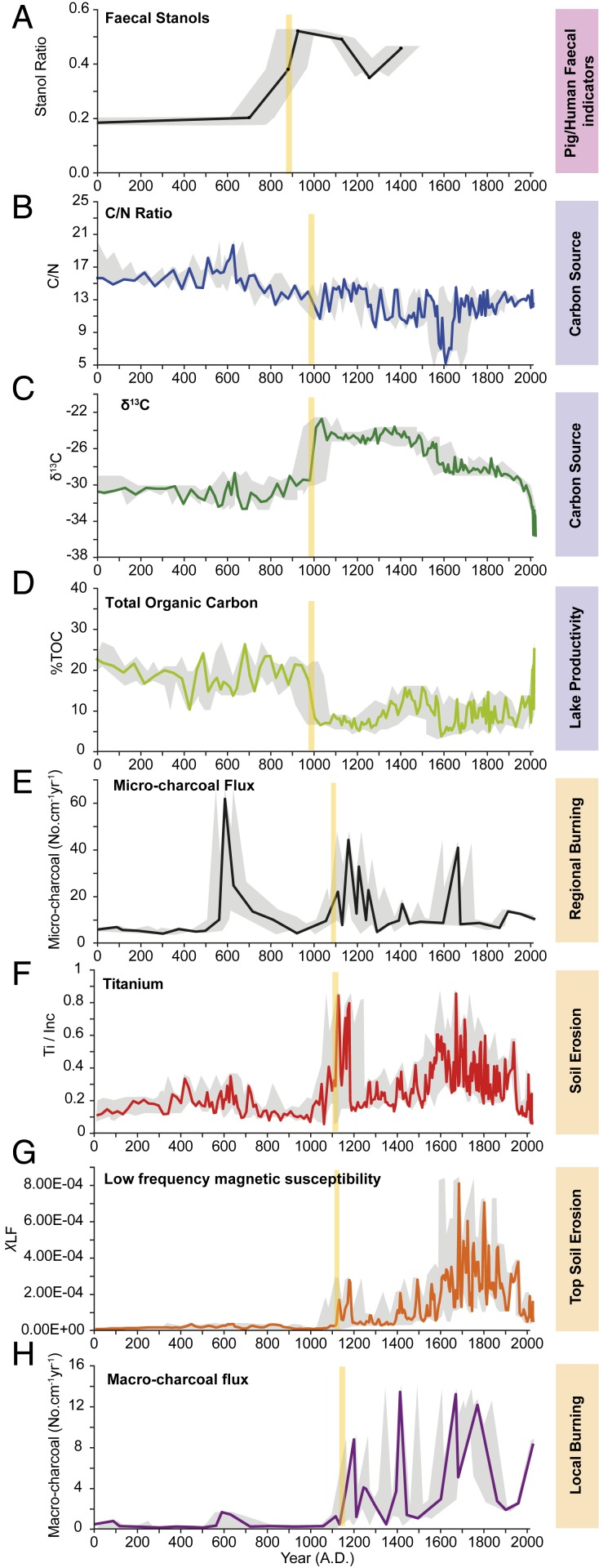
Proxy indicators from Lake Te Roto for the presence of humans on the Atiu Island landscape. Yellow bars are the best estimates of human impacts based on either the first increase in values above background levels or rapid changes in proxy values (e.g., %TOC). Fecal sterol values (*A*) prior to 800 AD are all lower than the 0.20 threshold for confident identification of our target species (pigs and humans) (21). Carbon source (*B* and *C*) and total organic carbon (*D*) change around AD 1000 as inorganic soil in-wash (*F*, Ti/Inc.; *G*, χLF) increases following disturbance of the catchment soils, presumably due to local burning and clearance (*H*). Microcharcoal (*E*) shows a large peak before all other indicators, which we interpret as natural burning given the absence of, or minor changes in, all other proxies at that time. The gray region represents 2 SDs around the age model weighted mean, combined with 1 SD in measured proxy values. Brief interpretations of what each proxy represents are shown for clarity (*Right*).

With more than 6,500 y of near-continuous sediment deposition, the Lake Te Roto core provides an excellent record of natural variability in the prehuman period. Throughout much of the core there is little variation but four exceptional intervals (Table 1) during the last 2,000 y are focused on here (Fig. 2) (17). To test for a human presence, we analyzed sterol biomarkers that are commonly associated with mammalian fecal remains (20, 21) from intervals bracketing changes that were suggestive of anthropogenic disturbances (e.g., C/N ratios, macrocharcoal, Titanium normalized by incoherence scatter [Ti/Inc.], etc.). Fecal sterols were first identified at 215 cm. The age measurements obtained for SLMs drive the Bayesian modeling and constrain the depth interval for initial fecal markers to AD 800 to 1004 (2σ). These unequivocal indicators of mammalian feces (pig and/or human) predate changes in lake productivity and major catchment disturbance, suggesting early humans and/or pigs associated with human arrival visited this significant water source but had negligible impacts on the immediate lake environment.

**Table 1.** Summary of Lake Te Roto (Atiu) proxy sequence (settlement phases after ref. 35)

Environmental phase	Measured proxy	Inferred variable	Sediment depth, cm	Bayesian modeled 2σ AD age range (midpoint)	Settlement phase (age estimate)	Hydroclimate Samoa/Atiu SCIs
Phase 1: regional disturbance	Microcharcoal	Regional burning	228	546 to 624 (585)	Natural variability or early discovery? (c. AD 600)	Wetter/wet
Phase 2: exotic species arrive	Fecal sterols	Presence of mammalian (pig/human) feces	215	800 to 966 (883)	Discovery (c. AD 900)	Drying/dry
	Fecal sterols	Presence of mammalian (pig/human) feces	212	828 to 1004 (930)		
Phase 3: changes in lake productivity and carbon sources	C/N	Lake carbon source	207	885 to 1123 (1004)	Colonization (c. AD 1000)	Dry/wetting
	% total organic carbon	Lake productivity	207			
	δ^13^C	Lake carbon source	207			
Phase 4: major catchment disturbances	Microcharcoal	Regional burning	196.5	1002 to 1210 (1106)	Establishment (c. AD 1125)	Wetting/wet
	Titanium (Ti/Inc.)	Soil erosion	196	1007 to 1219 (1113)		
	Low-frequency magnetic susceptibility (χLF)	Soil erosion	195	1010 to 1228 (1119)		
	Macrocharcoal	Local burning	188.5	1094 to 1214 (1154)		

**Fig. 3.**
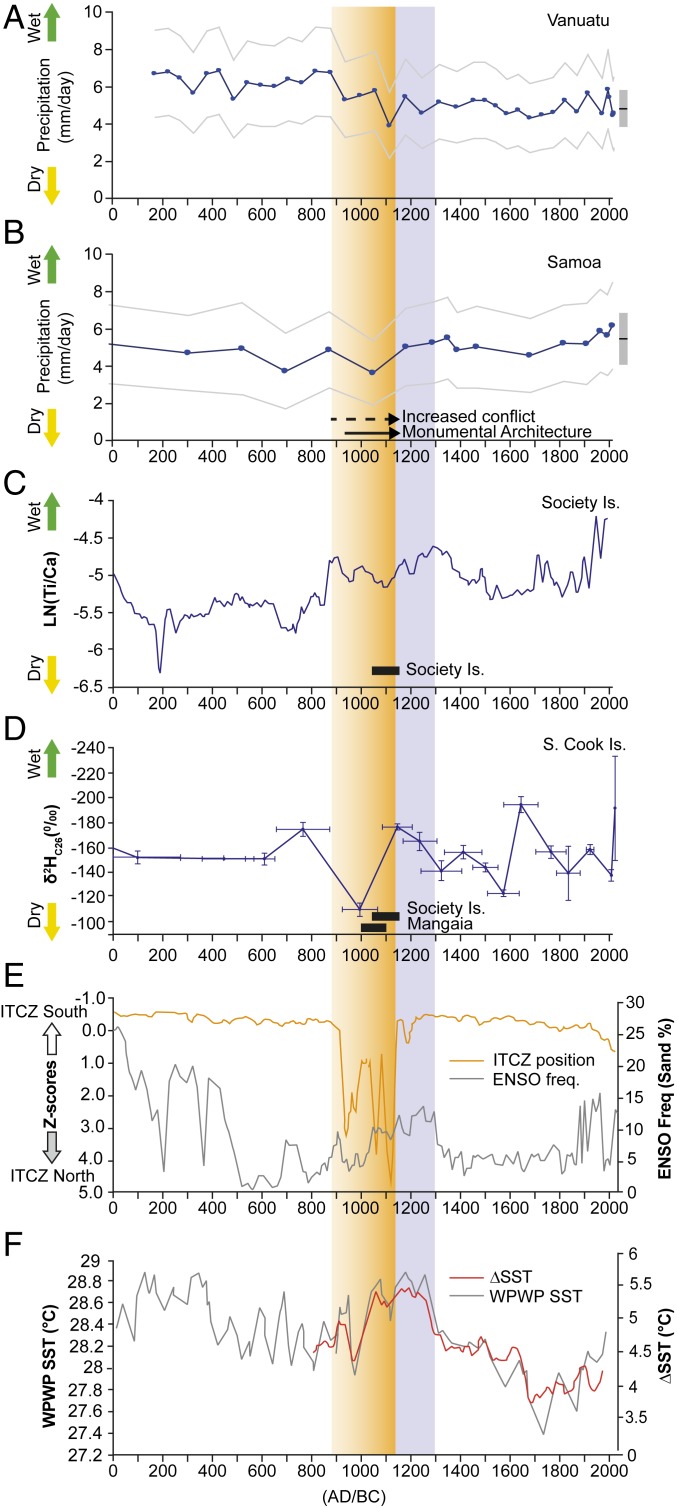
Reconstructed precipitation (rates and wet:dry trends) for west-to-east Pacific Islands in *A* to *D* (*C*, Society Islands data are based on ref. 27). In *A* and *B*, pecked lines denote uncertainty bounds around estimated precipitation, while gray bars with a black horizontal line (*Right*) show mean ± uncertainty for contemporary satellite-based [GPCP (28)] precipitation. The solid and dashed arrows in *B* represent periods of societal change in Samoa (29). Movement of the ITCZ (30) and ENSO frequency (31) are shown in *E*. Reconstructed SSTs in the West Pacific Warm Pool (WPWP) (32) and Pacific zonal SST gradients (33) are in *F*. Archaeological dates for the Southern Cook and Society islands are shown as horizontal black bars (*C* and *D*) while dates for Atiu (this study) are shown as a graded gold column from arrival c. AD 800 to 1000 through established settlement from c. AD 1125. The gray column denotes the later period of Polynesian expansion into remote eastern Polynesia including Rapa Nui c. AD 1150 to 1300 (34).

**Fig. 4.**
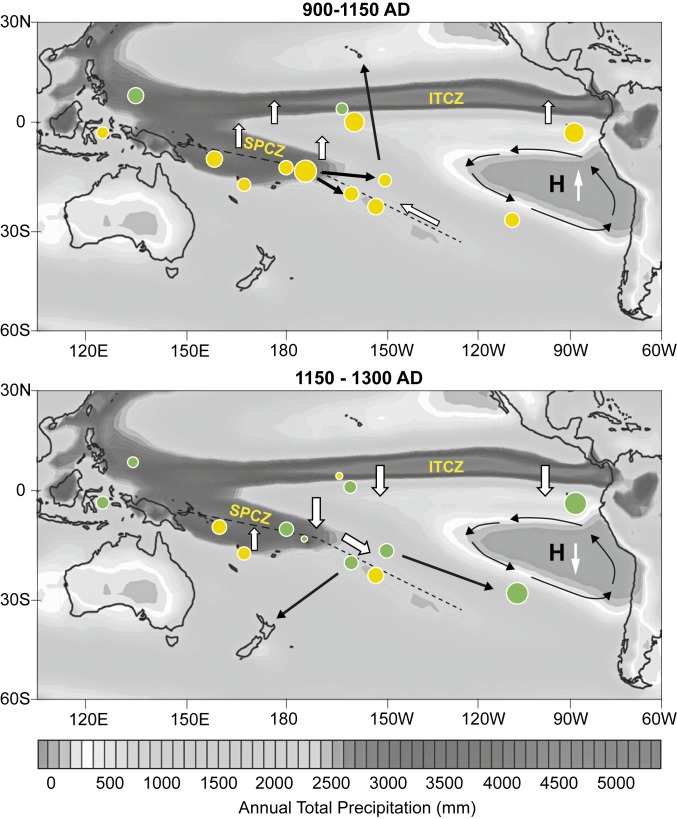
Synthesis of climate changes over the period of initial human migration into the East Polynesian gateway Islands c. AD 800 to 1125, and during the period of expansion into Rapa Nui and New Zealand c. AD 1150 to 1300 (corresponding to the gold column in Fig. 3). Circle colors show drier (yellow) and wetter (green) climate while sizes of circles are proportional to the magnitude of change. White arrows show movement of the ITCZ and SPCZ, and black arrows show the known extent of Polynesian voyaging. Initial migration east occurs during a regional change in climate as the ITCZ and SPCZ migrate north, while the latter contracts and weakens resulting in drought across the south Pacific Islands. Post c. AD 1150 the ITCZ moved south again and the SPCZ axis probably moved south and extended, providing better conditions for settlement on the more isolated and smaller islands of the eastern Pacific. Background colors show mean annual precipitation (GCGP 1976 to 2013). Dashed lines show the average axis of SPCZ precipitation over this latter period. Sources used for this figure are in *SI Appendix*, Table S4.

BACON 2.2 (19) was used to construct the Bayesian age model using ^14^C samples from stratigraphic depths at which proxies exceed background values or show a rapid rate of change (Fig. 2). Conventional ^14^C ages calibrated using ShCal13 (51). Hydroclimate in Samoa and Atiu (SCIs) is inferred from proxy climate data (lipid biomarkers and Ti/Inc.) from Lake Lanoto‘o and Lake Te Roto (Figs. 3 and 4). Drought in Samoa coincides with discovery (phase 2) and colonization (phase 3) on Atiu. Local hydroclimate on Atiu became increasingly wet toward the end of phase 3 (colonization) and leading into phase 4 (establishment).

Seven other proxies recovered from distinct lamina between 215 and 188.5 cm inform on subsequent changes in lake productivity and the onset of consequential catchment disturbances (Fig. 2 and Table 1). These include C/N ratios and δ^13^C of organic carbon, which indicate the organic matter is from a terrestrial carbon source, and total organic carbon, which is a measure of lake productivity (22, 23). Consequential changes in these three proxies are bracketed by ^14^C analysis of SLMs from 215 cm (c. AD 686 to 961, 2σ) and a second sample from 182.5 cm (c. AD 1047 to 1274, 2σ). The BACON Bayesian model narrows this time interval to c. AD 1004 (885 to 1123).

Other analyses include determination of micro- and macrocharcoal [regional and local burning, respectively (24)], titanium (Ti/Inc.), and low-frequency magnetic susceptibility (X_LF_), the latter two measures of soil erosion (25, 26). From a 196.5-cm depth, after c. AD 1106 (1002 to 1210) (BACON), changes are apparent in all seven proxies. Macrocharcoal density reaches a maximal peak, along with soil erosion proxies indicative of increased catchment disturbance. At the same time, leaf wax biomarker hydrogen isotopes identify this as a period of enhanced precipitation in the SCIs (Fig. 3*D*), conditions that would have favored agricultural investments and increased surface runoff. Notably, this time interval also coincides with direct archaeological evidence for human settlements throughout the SCIs (*SI Appendix*, Fig. S2).

Although the absolute chronology of three proxies cannot be unambiguously determined on the available AMS ^14^C data, three phases of disturbance can be identified on stratigraphic grounds and are assigned by the Bayesian analysis to between the 9th and early 13th centuries AD (Table 1, phases 2 to 4). Notably, values for all of these indicators, except microcharcoal, are higher in phases 2 to 4 than at any point in time in the c. 6,550-y continuous record (18). This, in conjunction with the human and/or pig fecal sterol evidence, leaves little doubt that phases 2 to 4 reflect human activity on the island.

Changes are also apparent around the 6th century AD (phase 1) but could reflect nonhuman processes. There is a peak in microcharcoal, accompanied by a small increase in macrocharcoal and a spike in C/N ratios between 228 and 216 cm (Fig. 2 and Table 1). Although this could derive from human activity on Atiu Island (17), fecal sterols are lacking and there is no direct archaeological evidence to support this interpretation. Given that microcharcoal can travel considerable distances, these changes could be indicative of burning elsewhere in the archipelago, or even farther afield.

Comparing the Lake Te Roto results with archaeological records from other Southern Cook Islands, the phase 2 fecal sterol records potentially predate archaeological evidence of human occupation by as much as 200 to 300 y (*SI Appendix*, Fig. S4). Phase 3 changes in C/N, total organic carbon, and δ^13^C point to more consequential human activities in the Lake Te Roto catchment, which predate secure archaeological indicators from Atiu Island. However, at Tangatatau Rockshelter (Mangaia Island), a chronological sequence places initial human visitation possibly as early as the 11th to 12th centuries based on U-Th dating of coral tools (AD 1011 ± 5.8 and 1167 ± 12) from earlier strata (3, 4). A single result from Aitutaki Island to the north indicates late 11th-century human activity (5). As a whole, the available archaeological records are consistent with the small-scale, low-impact human activities suggested by the Lake Te Roto phase 3 proxies and fully supportive of the phase 4 interpretations (*SI Appendix*, Fig. S4).

## The Climatic Context of Eastward Dispersals

The three lake archives provide a regional perspective on climate variability over the last 2,000 y. Particular attention is drawn to the period AD 900 to 1200, a likely window of East Polynesian colonization (Fig. 3). The Vanuatu precipitation record derived from algal lipid hydrogen isotope (δ^2^H_dinosterol_) measurements (*Materials and Methods*) identifies the period between c. AD 0 and 900 as the wettest in the total lake sediment sequence (average 6.3 ± 2.2 [average uncertainty] mm/d). Thereafter, conditions become increasingly dry over the period between AD 1047 and 1222 (2σ), producing the driest values (average 3.9 ± 1.8 [average uncertainty] mm/d) in the entire 2,000-y period; notably, contemporary estimated values for the period 1976 to 2017 average 4.9 ± 1.9 (average uncertainty) mm/d. The Samoan δ^2^H_dinosterol_ record is similar to Vanuatu, but variability is less pronounced given its location within the main axis of the SPCZ. The period between c. AD 0 and 700 has an average precipitation of 5.0 ± 2.2 (average uncertainty) mm/d. Thereafter, two particularly dry periods occur: one estimated at 3.8 ± 2.0 mm/d at c. AD 700 (AD 528 to 872, 2σ), and a second more precisely defined dry interval estimated at 3.7 ± 1.7 mm/d, which occurred around AD 1000 (AD 892 to 1036, 2σ) (Fig. 3*B*). By comparison, contemporary estimated values for the period 1963 to 2013 average 5.9 ± 2.2 (average uncertainty) mm/d. In the Lake Te Roto record, the driest period in the last 2,000 y as indicated by the leaf wax record (Fig. 3*D*) occurred around c. AD 990 (AD 923 to 1058, 2σ).

The period of drier hydroclimate coincident with the arrival of humans in the SCIs is identified in all three lake sediment cores. The reductions in precipitation are well below contemporary averages and are of the same magnitude as contemporary ENSO precipitation anomalies (La Niña−El Niño), which result in drought conditions on Vanuatu, Samoa, and Atiu. We posit that these marked precipitation anomalies were of sufficient magnitude as to adversely impact agricultural productivity and freshwater resources relative to subsequent and previous centuries, and are indicative of a prolonged drought in this region of the Pacific.

## Discussion

Our sediment core results from the Southern Cook Islands provide evidence of anthropogenic activities from time intervals that predate archaeological evidence from across the archipelago. The lake’s laminated gyttja allows for the identification of four sequential phases of unprecedented environmental disturbance, while ^14^C analyses provide an absolute chronology of disturbances between 800 and 1225 AD: regional burning (possibly anthropogenic) in the 6th century AD (phase 1); the arrival of exotic mammals (humans and/or pigs) (phase 2: c. AD 900); changes in the lake’s productivity and organic carbon sources roughly a century later (phase 3: c. AD 1000); and major catchment disturbances within the following century (phase 4: c. post AD 1100) (Table 1). These empirically defined phases resonate with conceptual models of island settlement, which differentiate between island discovery, colonization, and establishment (10, 35). New islands may be located during strategic exploratory voyages or as accidental landfalls, and in turn become localities of resource extraction (e.g., birds, marine resources) and/or waystations from which to conduct further exploration. Given the distances between settled islands in the west and those of the vast East Polynesian region, provisioning stations (especially those with fresh water) may have been crucial in the eastward expansion process. The Lake Te Roto SCI record (Fig. 2 and Table 1) suggests populations from West Polynesia began exploratory probes along the margins of East Polynesia into the SCIs and possibly other proximate archipelagos such as the Societies, well before full-fledged colonizing expeditions were launched.

More modest changes in lake productivity followed in the next 1 to 2 centuries, potentially signaling the arrival of colonizing parties. Archaeologically, 11th-century colonists are suggested on the neighboring island of Mangaia, where Polynesian rats (*Rattus exulans*) and coral tools date to this interval (4). By the 12th to 13th centuries, major lake catchment disturbances on Atiu are indicated, including vegetation burning and soil erosion. Both suggest forest clearance and probably agricultural activities—processes that are consistent with established, permanent, and larger settlements. These latter activities are archaeologically well-demonstrated on islands to the north (Aitutaki) and south (Mangaia) of Atiu (*SI Appendix*, Fig. S2), indicating human populations had dispersed across the SCI chain by this time.

The Lake Te Roto findings also resonate with reconstructions of synoptic wind patterns and Polynesian voyaging to the region’s margins (Rapa Nui, New Zealand) (36). Goodwin and colleagues (36) identify key sailing windows, when “the centennial mean climate pattern resembles a shift to the Central Pacific (Modoki) El Niño pattern, with … anomalous westerly wind fields (trade wind reversals) over the Central Pacific.” Relevant to the Te Roto findings, they identify sailing windows between West Polynesia and the SCIs around AD 860 to 900, when mammalian (pig/human) fecal sterols make their initial appearance. Explorations of the kind suggested here may have been planned to take advantage of such seasonal and interannual wind shifts, as these would have facilitated easy and safe homeward voyages (10). A second key sailing window is identified at c. AD 1040 to 1060, coinciding with increases in lake productivity and phase 3 colonization (Table 1).

The lake sediment archives from our three study sites, in combination with other published records, allow for the reconstruction of paleoclimatic conditions over the last 2 millennia and provide context for the trends observed at Lake Te Roto. The initial phase of East Polynesian exploration (Fig. 3) as indicated by the SCI records (c. AD 800 to 1000) is characterized by an abrupt reduction in zonal sea surface temperature (SST) gradients and cooling of the West Pacific Warm Pool (Fig. 3*F*) (33). The central Pacific Intertropical Convergence Zone (ITCZ) moves north c. AD 900 (30), while far eastern Pacific El Niño frequency and magnitude remain low (Fig. 3*E*) (31). Model predictions support a northwest movement, contraction, and weakening of the SPCZ as the SST zonal gradient and absolute SSTs decrease (37)—conditions consistent with the prolonged regional drought suggested by our core proxies (Fig. 4*A*).

From AD 1000 to 1150, a rapid increase in SSTs and strengthening of zonal SST gradient (Fig. 3*F*) correspond to strengthening of the eastern SPCZ, enhanced by moisture transfer as the southeast Pacific high strengthens, as indicated by drought on Rapa Nui (34). In the Atiu sediment record (Fig. 3*D*) and also in the Society Islands (27) (Fig. 3*C*), this is evidenced by concomitant precipitation increases. At the same time, conditions remained drier on Samoa and Vanuatu, reaching a 2,000-y minimum c. AD 1050 to 1100 (Fig. 3 *A* and *B*), consistent with a generally more northerly location of the main SPCZ axis. At this point in time, precipitation values in Samoa were reduced by 38% below contemporary values, presumably stressing critical food and water resources (see also refs. 13 and 27). This period of unprecedented drought coincides with the increased disturbance indicators on Atiu described above (Fig. 2 and Table 1) which, when paired with human and/or pig fecal markers, strongly suggests human activity on the island.

From c. AD 1150 to 1300, the period in which the furthest regions of Polynesia were colonized, the climate of the South Pacific changes (Figs. 3 and 4*B*). SSTs in the West Pacific Warm Pool and zonal SST are high, and the central Pacific ITCZ moves south or expands (Fig. 3*E*) (wetter in Kiribati c. AD 1150) (Fig. 3*E*). Under these conditions the SPCZ typically moves south and extends southeast and precipitation intensifies and becomes more variable with increasing ENSO frequency (37), resulting in the observed increases in precipitation inferred from regional proxy climate data (38) (Fig. 4*B*). ENSO frequency is higher at this time (Fig. 3*E*), forcing increased variability in precipitation but also opening sailing windows to Rapa Nui and New Zealand (36). Dates for the arrival of people on Rapa Nui [c. 1150 to 1300 AD (21, 34)] coincide with this period—the voyages perhaps being supported by increased precipitation at this time (13).

Our climate data coincide with evidence from Samoa and Tonga for marked demographic changes, growing social inequality, and intergroup competition from as early as the 5th century AD (29, 39). By the 9th century AD, both traditional histories and archaeological records suggest emergence of the powerful Tongan maritime chiefdom and its hegemonic expansion across West Polynesia over the next few centuries (39). The intersection of these developments with the prolonged, regional drought indicated in our cores was undoubtedly consequential in eastward explorations and ultimately outmigrations from the West Polynesian heartland [or possibly elsewhere (40)]. The Lake Te Roto records suggest that rather than precipitous or haphazard departures, settlement of the SCIs was an incremental, multiphased process that potentially involved the accumulation of critical environmental knowledge over several generations. These findings have implications for current thinking about the timing, character, and pulse of East Polynesian settlement as a whole.

## Materials and Methods

Cores were collected from all lakes using a combination of UWITEC gravity coring to capture the top of the sediment including the sediment–water interface and piston coring (GeoCore) to recover subsequent overlapping sequences of sediment. All cores were kept intact and stored in airtight tubes during transport and placed in cold storage (+4 °C). In the laboratory, cores were split longitudinally and underwent low-frequency magnetic susceptibility analysis and micro X-ray fluorescence (μXRF) analysis before being subsampled at contiguous 1-cm intervals for loss on ignition (LOI). We cross-correlated the individual cores using the continuous data (LOI, magnetics, μXRF) to produce single master core sequences.

In all three lakes, we reconstructed hydroclimate using hydrogen isotope ratios of lipid biomarkers (δ^2^H_lipid_ = ([^2^H/^1^H_smpl_]/[^2^H/^1^H_std_]) − 1)*1000, where std is Vienna standard mean ocean water). Sediment subsamples (1 cm thick) were removed from split cores or from field-sectioned material and transferred to combusted glass vials, frozen, and then freeze-dried. Lipids were purified and quantified following procedures detailed in *SI Appendix* and refs. 41 and 42. For Lake Emoatul (Vanuatu) and Lake Lanoto‘o (Samoa), precipitation (mm/d) was calculated with the δ^2^H_dinosterol_−GPCP (Global Precipitation Climatology Project) core top calibration detailed in ref. 41 and *SI Appendix*. Algal lipids obtain all of their lipid hydrogen from lake water, which in turn responds isotopically to increases and decreases in precipitation and evaporation. For Lake Te Roto, Atiu (SCIs), we used high-chain-length fatty acids (C_26_) to determine hydrogen isotope ratios of terrestrial plant leaf waxes to avoid complications of ocean water connections to the lake and associated salinity effects on aquatic lipids that may not coincide with changes in hydrology (43, 44). The hydrogen isotopic composition of plant leaf waxes (δ^2^H_lw_), including the long-chain n-alkyl compounds that comprise the waxes, is largely controlled by the hydrogen isotopic composition of a plant’s source water (soil water) δ^2^H and relative humidity (45, 46).

For the Lake Te Roto core, eight proxy indicators were analyzed. We used microcharcoal (<125 μm) and macroscopic (>125 μm) charcoal to define the presence of burning on the landscape (47). To assess changes in soil erosion within the catchment, we measured magnetic susceptibility and titanium (25, 26). Lake productivity and in-wash were tracked using C/N, δ^13^C, and total organic carbon (TOC) (22, 23). We normalized the Ti terrigenous proxy data by the Compton/incoherent scatter signal from the μXRF ITRAX scanner to account for changes in water and organic matter content (48).

To confirm the presence of mammals (pigs and/or humans), we measured fecal sterol ratios (20). Lipid biomarker analysis followed standard protocols (49) (*SI Appendix*). Sampling for fecal sterol ratios was concentrated at sediment core intervals, where initial anthropogenic disturbance was suggested by macrocharcoal, magnetics, and Ti/Inc. values. Given that steroids (in particular 5β-stanols) have low water solubility and are mainly adsorbed to particulate organic matter, they are not prone to leaching (50). Further details on all methods are found in *SI Appendix*.

### Data Availability Statement.

All data supporting this study are contained in Dataset S1.

## Supplementary Material

Supplementary File

Supplementary File
