# Supplementary material for: Human settlement of East Polynesia earlier, incremental, and coincident with prolonged South Pacific drought
Source: Proc Natl Acad Sci U S A. 2020 Apr 6;117(16):8813–9. doi: 10.1073/pnas.1920975117 (PMC7183181; doi:10.1073/pnas.1920975117)
Supplement: Supplementary File [file pnas.1920975117.sapp.pdf]

PNAS
www.pnas.org

Supplementary Information for

Human settlement of East Polynesia earlier, incremental, and coincident with prolonged South Pacific drought

David A. Sear, Melinda S. Allen, Jonathan D. Hassall, Ashley E. Maloney, Peter G. Langdon, Alex E. Morrison, Andrew C.G. Henderson, Helen Mackay, Ian W. Croudace, Charlotte Clarke, Julian P. Sachs, Georgiana Macdonald, Richard C. Chiverrell, Melanie J. Leng, L M Cisneros-Dozal, Thierry Fonville

Email: d.sear@soton.ac.uk

This PDF file includes:

Supplementary text
Figures S1 to S4
Tables S1 to S4
Legends for Datasets S1
SI References

Other supplementary materials for this manuscript include the following:

Datasets S1 Bayesian Model Code, and the Datasets

Supplementary Information

Core Extraction Methods and Description

To capture the full sediment sequence we deployed a UWITEC gravity-type corer with core catcher and a barrel length of 60 cm; this allowed us to capture the sediment-water interface and most recent sediments. Sediment loss was avoided with the core catcher that seals the bottom of the core *in situ* before withdrawal through the water column. For subsequent sediment depths a 120 cm-long Livingstone-type corer (Geocore) was employed with both 5 cm and 3 cm core diameters to retrieve overlapping cores. All cores were kept intact and stored in an airtight tube during transport and when placed in cold storage (+4 °C). The cores were split longitudinally and underwent low-frequency magnetic susceptibility analysis and micro X-ray fluorescence (μXRF) analysis before being sub-sampled at contiguous 1 cm intervals.

A sequence of overlapping cores was obtained for Lake Te Roto to an overall depth of 783 cm. The sequence is laminated gyttja throughout, alternating between light grey and black lamina. (Fig S1). In Lake Lanoto‘o[1], a sequence of overlapping cores was obtained to an overall depth of 302 cm. There is some visual stratigraphic change throughout the sequence, primarily alternating between two units of very dark brown gyttja and strong brown silicilastic sediment. In Lake Emoatul, a sequence of overlapping cores was taken from the deepest part of the lake (7.1m) giving an overall sediment depth of 3.40m. Visual stratigraphy showed laminations of alternating dark brown lake gyttja and darker brown-black organic rich layers. The lower 10 cm of the core were grey clay, which prevented further penetration and sampling. All cores were correlated using LOI, magnetic and Itrax data, with core depths then re-mapped onto a single depth model that has been used for age/depth modelling (Figure S2).

Core Chronometric Analyses

To build precise chronologies for upper core sediments (top 0.5 m), ^{210}Pb was measured (via its granddaughter ^{209}Po) using double aqua regia acid leaching of sediment, ^{209}Po spiking, and auto-deposition onto silver discs to determine ^{210}Pb activities using alpha spectrometry[2]. A chronology was generated from excess ^{210}Pb activity data for each core by applying the constant flux:constant sedimentation (CF:CS) model.

The age-depth models are constrained by ^{210}Pb, ^{137}Cs and ^{14}C AMS ages and in Vanuatu, a single dated tephra. The ^{14}C ages were calibrated using the SHCal13 atmospheric curve[3]. BACON, the software used for Bayesian age-depth modelling, negates the effects of outlying dates because the ages are modelled using a student-t distribution with wide tails[4]. Given the greater confidence generated by ^{210}Pb, ^{137}Cs , tephra and ^{14}C SLM sample dates, these ages were assigned more narrow Gaussian error distributions reflecting their greater reliability. Thus, in the final Bayesian models, age measurements are constrained by the more robust short-lived materials. All ages throughout the article refer to modelled ages based on the resulting age:depth model. The model is provided in the data link at the start of this SI.

The Lake Emoatul (Vanuatu) age model is based on seven AMS ^{14}C analyses on SLM, specifically terrestrial monocot leaf fragments and in one case a seed, from throughout the core and 33 ^{210}Pb analyses from the upper 0.5 m. A distinctive tephra geochemically linked to the large, local Kuwae eruption of c. AD 1457 provides an additional chronological constraint. Final age model uncertainties around the proposed period of East Polynesia colonization (c. AD 900-1200) were ±85 yrs (Table S1, Fig. S2). The age model for the Lake Lanoto‘o (Samoa) core is derived from 14 AMS ^{14}C analyses on bulk sediments, three on short lived leaf fragments (SLM), one on an unidentified species of wood, along with 21 ^{210}Pb dates from the upper 0.5 m of the core. The resulting calibrated age model provides 2σ uncertainties of ±59 yrs around the presumed period of migration into East Polynesia (Table S2, Fig. S2).

An absolute age model for Lake Te Roto (southern Cook Islands) is provided by 24 ^{210}Pb dates (from top 0.5 m) and 20 AMS ^{14}C dates from throughout the core (see Table S3, Figs. S1 and S2).The final age model uncertainties associated with the period of likely anthropogenic disturbance are ±140 yrs. In the Te Roto core, terrestrial monocot leaf fragments were preferentially sought for ^{14}C dating, but were not always available, requiring some bulk sediment analyses (see Table S3, Figure 2). Results on bulk sediment are typically older than those on equivalent-depth SLM, by between 30 to 284 radiocarbon years. Given the finely laminated nature of the sediments, we suggest this does not reflect sedimentary disturbances, but rather the inclusion of older carbon in bulk sediment samples as is typical when dating bulk materials. In light of this, while all dates were included in the final Bayesian model[3], higher weights were given to SLM results (Fig. S2).

Archaeological Settlement Chronologies

Archaeological research on Aitu has historically focused on the island's surface architecture and caves. In the 1960s, Roger Duff[5] and Michael Trotter[6] recorded 32 sites, including marae (temple sites), caves with human burials, and habitation areas. Steadman[7] explored some 20 caves on Atiu in an effort to document the island's indigenous avifauna—most of these showed little sign of human habitation. Steadman speculated that human arrival on Aitu was probably contemporaneous with that on Mangaia Island, noting that the makatea on the former was somewhat less rugged. Kurashina, Stevenson and Sinoto[8] mapped and stabilised some of the island's marae.

In 1987 Allen and Steadman tested a habitation terrace (designated Site 33) on the margins of a swamp to the north of Lake Te Roto that had been used for taro cultivation in the past. A single charcoal sample from the lowest occupation layer returned a conventional ^{14}C age of 280 ± 80 (Beta-27438). Calibrating to c. AD 1460-1950 (2σ; median AD 1662), the result suggests use of this swampy area pene-contemporaneous with the late prehistoric spikes in burning and soil erosion recorded in the Lake Te Roto core. This occupation layer was overlain by a thick clay deposit that included a considerable number of land snails, suggesting removal of native forest or shrub at this time.

Although no early settlement sites have been formally identified on Atiu, Walter[9,10] excavated a 14th century village complex on nearby Ma 'uke, an island in regular contact with Atiu at the time of European arrival. Walter[10] observed that Atiu is a comparatively high and well-watered island. Its lake, extensive swampland suitable for wetland taro cultivation, and patches of good volcanic soil might have made it a more attractive island for early settlement relative to nearby Ma'uke and Mitiaro.

Most recently, bio-archaeological research was carried at the Rimu Rau burial cave by Angela Clark and colleagues[11]. This sizable limestone cave complex, along with two others, are in close proximity to Lake Te Roto. Clark and team identified more than 600 individuals, interments traditionally associated with a major battle between rival tribes. Although Clark et al.[11] attempt to make a case that the interments date to early settlement period, none of the cave materials were dated and distinctive artefacts that might have suggested relative ages were lacking. Given the oral traditions, it seems more likely that the Rimu Rau interments are largely, if not solely, late prehistoric in age.

To compare the results from Lake Te Roto with current archaeological chronologies, we re-calibrated the extant corpus of ^{14}C analyses from the earliest archaeological contexts for the southern Cook Islands of Mangaia and Aitutaki (Fig S3). This dataset includes samples of short-lived plant material and bones of the introduced Polynesian rat (*Rattus exulans*) recovered from Zones SZ1B and SZ2 at the Mangaian site of MAN-44[12,13]. The Aitutaki samples (mostly coconut endocarp) come from the earliest contexts at two mainland sites located on Aitutaki's west coast (Ureia/AIT-10 and Hosea/AIT-50), and the earliest occupation in the Moturakau Rockshelter (MR-

1) located on a small offshore islet[14,15]. The ^{14}C results were calibrated using OxCal ver. 4.3[16] and the SHCal13 atmospheric curve[3].

Micro- and Macro-charcoal Analyses

Micro- and macro-charcoal have been shown to reflect regional and local burning, with the latter frequently interpreted in Pacific island sediment archives as an indicator of the arrival of humans[17]. Samples were analyzed in the Te Roto (Atiu) core for both microscopic (<125 µm) and macroscopic (>125 µm) charcoal, following standard protocols[18,19]. Micro-charcoal samples were mounted on slides using silicon oil and counted under a high-power microscope at 600x magnification. Macro-charcoal samples were placed into a grooved Perspex sorting tray (Bogorov tray) with a groove 5 mm deep and 5 mm wide and counted under a stereo microscope. Samples were initially scanned at 20x magnification with charcoal identification confirmed at 40x magnification. A minimum count sum of 500 items (i.e., the sum of microscopic charcoal particles and *Lycopodium* exotic markers) per sample was used. For macroscopic charcoal (>125 µm), all fragments present within the sample were counted. Concentrations (particles/cm^3) were multiplied by sediment accumulation rate (cm/yr^{-1}) to obtain charcoal accumulation rates (CHAR; particles cm^{-2} yr^{-1}).

Faecal Sterol Analyses

Faecal sterols have been used to address questions relating to prehistoric animal husbandry, human palaeodemography, and the arrival of humans in virgin landscapes[20,21,22]. However, archaeological applications have been largely limited to temperate environments[21]; the present analysis is the first application to tropical Polynesia, a region where pigs, dogs, and rats are non-native, having been introduced by human colonists. Faecal sterols often enter lake environments via runoff and thus typically inform on human activities within the wider lake catchment[22]. Faecal Sterols (including faecal sterols (5β-stanols)) have low water solubility and are mainly adsorbed to particulate organic matter[20]; consequently they are not prone to leaching[23].

For the Te Roto lake core, 9 samples obtained between 426 cm (3695 cal BP) and 144 cm (c. AD 1404) sediment depth were analyzed for faecal sterol ratio values representing pig and human excrement', of which 7 were in the period c. AD 0-1404 (Fig 2). All samples earlier than c. AD 883 (800-966 2σ) were below the sterol ratios indicative of the presence of pigs or humans[20]. Analyses were conducted following standard protocols[24]. Briefly, 10 µl of androstanol (0.1 mg/ml) was added as an internal standard to each sample of approximately 1 g of dried, homogenized sediment. Lipid compounds were extracted with solvents (DCM:MeOH, 3:1) using Microwave Assisted Extraction[25], saponified and separated into neutral and acid fractions using aminopropyl SPE columns. The neutral fraction of each sample was then separated using silica gel column chromatography to isolate the sterol fraction. The sterol fraction was trimethylsilylated using 30 µl N,O Bis(trimethylsilyl)trifluoroacetamide (BSTFA)/ trimethylcholorosilane (TMCS) (99:1 v/v) and heated at 70 °C overnight. Excess BSTFA-TMCS was removed by drying gently under nitrogen. Samples were dissolved in 50-100 µl of ethyl acetate prior to gas chromatography-flame ionization detection (GC-FID) and gas chromatography-mass spectrometry (GC-MS) analysis. GC-MS analyses were performed on an Agilent 7890B GC injector (280 °C) linked to an Agilent 5977B MSD (electron voltage 70eV, source temperature 230°C, quad temperature 150°C multiplier voltage 1200V, interface temperature 310°C) in full scan mode (50-600 amu/sec). Separation was performed on an Agilent fused silica capillary column (HP-5, 60 m, 0.25 mm ID, 0.25 um df), with Helium as a carrier gas. The sample (1µl) was injected in splitless mode (1 min splitless time). Sterol derivatives were analyzed using the following temperature program: 50 °C (held for 2 min) to 200 °C at 10°C min^{-1} then to 300 °C at 4 °C min^{-1} and held for 20 min. GC-MS peaks were identified through comparisons with known mass spectra (NIST08[20]) and standards where possible. Analytes were quantified based on internal standards.

Plant Lipid Analyses

In all three lakes we reconstructed hydroclimate using hydrogen isotope ratios of biomarker proxies. In Emoatul (Vanuatu) and Lanoto'o (Samoa) we analyzed the algal lipid biomarker dinosterol according to the methods detailed in[26]. In Lake Te Roto (Atiu), the periodic lake-ocean connections precluded use of $\delta^2H_{dinosterol}$ since hydrogen isotope ratios in algal lipids vary with salinity[27] and changes in this lake's salinity may not be correlated with changes in hydroclimate, also variable contributions from different species of marine dinoflagellates and freshwater lake dinoflagellates could complicate hydroclimate interpretation. Instead we used hydrogen isotopes in terrestrial leaf wax lipids[28,29].

<u>$\delta^2H_{Dinosterol}$ (Lake Emoatul and Lanoto'o)</u>

Sediment subsamples (1 cm-thick) were removed from split cores or from field-sectioned material. Dinosterol was extracted, identified, quantified, and purified using 2-step column chromatography and HPLC following procedures detailed in[26,30]. Prior to HPLC, samples were acetylated using acetic anhydride with a known hydrogen isotopic composition (-123.8 ± 8.2‰). $\delta^2H_{dinosterol}$ values were measured via gas-chromatography isotope-ratio mass spectrometry (GC-IRMS) using instrument conditions outlined in[26]. The H_3^+ factor[31] was measured prior to every sequence and was 1.74 ± 0.02 ppm nA^{-1} during the 2.5 months that 6 sequences were run for Vanuatu and was 1.98 ± 0.33 ppm nA^{-1} during the 2 months that 4 sequences were run for Samoa. External standards of known hydrogen isotopic composition (Dr. Arndt Schimmelmann, Indiana University, http://mypage.iu.edu/~aschimme/compounds.html) were injected throughout each run. Any peak areas less than 11 Vs were disregarded to avoid size dependent δ^2H effects[32] and isotopic compositions were evaluated in the Isodat 2.0 software relative to calibrated H_2 reference gas. $\delta^2H_{dinosterol}$ values were corrected using the regression of known versus Isodat-reported *n*-alkane standard δ^2H values. $\delta^2H_{dinosterol}$ values were then corrected for hydrogen added during acetylation by a mass balance calculation as in[33]. For Vanuatu 40 samples were injected on average 3.4 times and had a pooled analytical uncertainty of 4.0‰, for Samoa 20 samples were injected on average 4.1 times and had a pooled analytical uncertainty of 7.2‰. Using the $\delta^2H_{dinosterol}$ values produced for Lake Emoatul (Vanuatu) and Lake Lanoto'o (Samoa), precipitation was calculated with the $\delta^2H_{dinosterol}$ - GPCP core top calibration[26] using equation (1):

$$Pp = [\delta^2H_{dinosterol} - b\,] / m \qquad (1)$$

where Pp is the paleoprecipitation rate (mm/day), $\delta^2H_{dinosterol}$ is a down core measurement of sedimentary dinosterol, b is the intercept of the regression (211 ± 15), and m is the slope of the regression (12.1 ± 2.6). Uncertainties were calculated using a Monte Carlo approach with 100,000 iterations with normally distributed errors from analytical uncertainty in $\delta^2H_{dinosterol}$ measurements plus calibration error in the slope (±2.6) and intercept (±15).

<u>Leaf Wax Biomarkers (Lake Te Roto)</u>

The hydrogen isotopic composition of plant leaf waxes (δ^2H_{lw}) including the long-chain *n*-alkyl compounds that comprise the waxes, is largely controlled by the hydrogen isotopic composition of a plant's source water δ^2H[35,28]. *n*-alkanoic acids are straight-chain hydrocarbons, with the main chain length, carbon number distributions and isotopic composition dependent on the source organism[36]. Since C_3 and C_4 plants fractionate meteoric water differently leading to changes in biomarker δ^2H values, we used higher chain length (C_{26}) terrestrial-sourced $\delta^{13}C$ biomarker records to determine hydrogen isotope ratios so as to avoid salinity affects potentially found in lake-based carbon sources[37,36].

Sub-samples were taken from cores and freeze-dried. For δ^2H determinations each sample was first dissolved in a solution of hexane which contained sacrificial compounds ethyldecanoate and pentadecane at ca. 0.3 mg/cm^3. Compound-specific δ^2H determinations were performed using a

ThermoScientific Trace 2000 gas chromatograph coupled to a ThermoScientific Delta V *via* a GCIsolink and Conflow IV interface. All analyses were conducted in duplicate. The instrument performance was evaluated and a H_3^+ factor calculated on a daily basis using H_2 reference gas (stable and <2 ppm/mV)[31]. Data were initially calibrated to two H_2 reference peaks injected directly into the ion source, before being normalized using the equation of a line from a plot of measured versus known δ^2H values for a standard suite of 15 *n*-alkanes (C_{16}-C_{30}; Mixture B3, Arndt Schimmelmann, University of Indiana) which was injected prior to every two sample runs. Peak heights <50mV identified on the GC-IRMS were below the cutoff and not considered to avoid size-dependent δ^2H effects[38]. Instrument error was typically less than 5‰, calculated using the same *n*-alkane standard. As the δ^2H for the esterification agent was not obtained, a correction for the addition of three Hs added during methylation to the C_{26} *n*-acid δ^2H value has not been undertaken for this site. As the trends will be the same, a qualitative interpretation of changes in the δ^2H values is sufficient to determine paleoenvironmental and paleoclimatic changes.

$\delta^{13}C_{TOC}$ plot within generalised C_3 terrestrial plant values, indicating that changes in δ^2H_{C26} are not a consequence of changes between C_3 and C_4 plants[29]. Moreover, δ^2H_{C26} values track changes in Ti/inc and XLF magnetics (both indicative of terrigenous inwash) such that more negative (less negative) δ^2H_{C26} are associated with higher (lower) Ti/inc and XLF (Fig S4). Thus we interpret changes in δ^2H_{C26} with qualitative changes in precipitation amount in Atiu for the period of record.

Regional Climate Proxies

For regional climate proxies we used published data from lake or swamp sites across the Pacific (Table S4). We converted all proxy data records for the past 2,000 years into normalized z-scores and averaged the values for two time periods[39]: AD 900 to 1150, the period of arrival and establishment in the southern Cook Islands (based on results from this study) and the Society Islands (see main text), and AD 1150 to 1300 the period in which much of the remainder of East Polynesia was colonized. We plotted the average z-scores in Figure 4 using graduated proportional circles in ARCMAP 9.2, color-coded to indicate wet and dry. We assume the values reflect relative magnitude of changes in wetness.

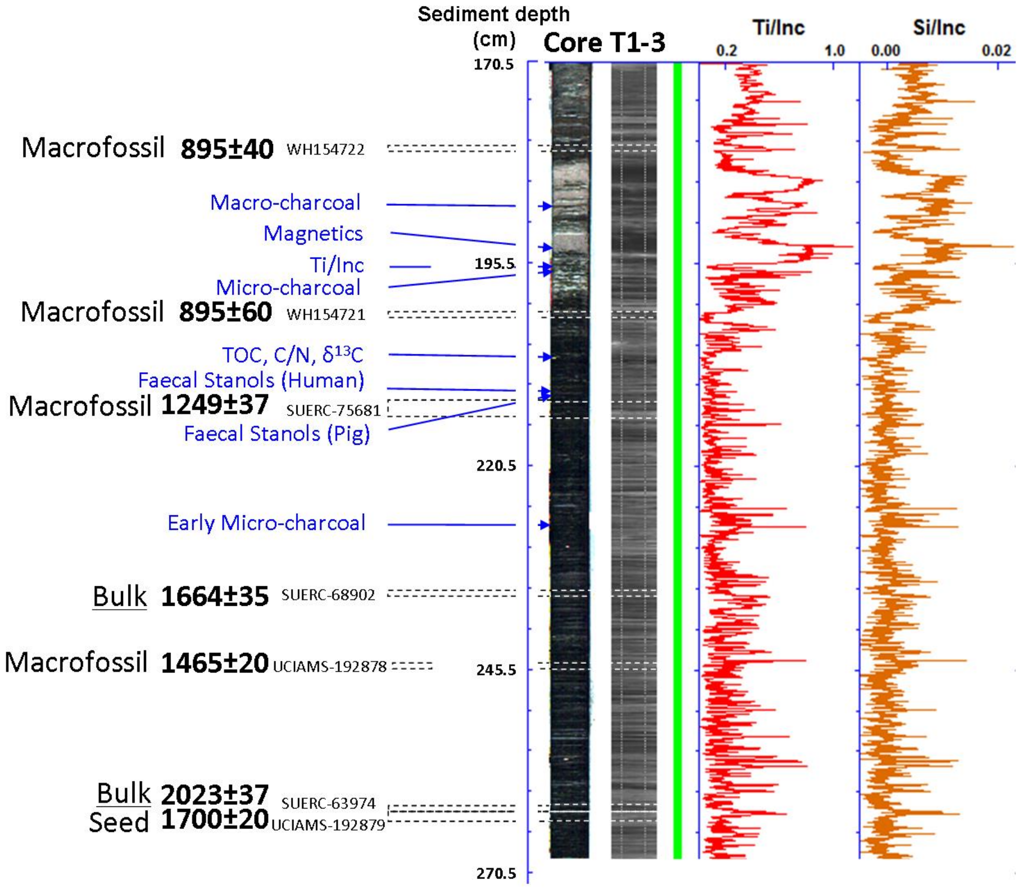

Fig. S1. Stratigraphy of the main Te Roto core (T1-3). The first two columns, optical and x-ray images of core T3-1, show the alternating light:dark laminations that indicate no disturbance or evidence of bioturbation. Coloured line graphs show the associated Titanium and Silica µXRF geochemistry. The position of AMS radiocarbon samples taken from this core are identified together with sample type, uncalibrated radiocarbon age and radiocarbon laboratory number (see Table S3 for further details). The additional radiocarbon sample from sediment depth 202.5 cm is not shown as it was sampled from a different core (T2-1). All macrofossils other than the one seed are fragments of monocot leaf fragments, tentatively identified as terrestrial grasses, but the species were undeterminable (see Table S3). Blue arrows denote the locations of the major changes in proxies as shown in Figure 2 of the main text.

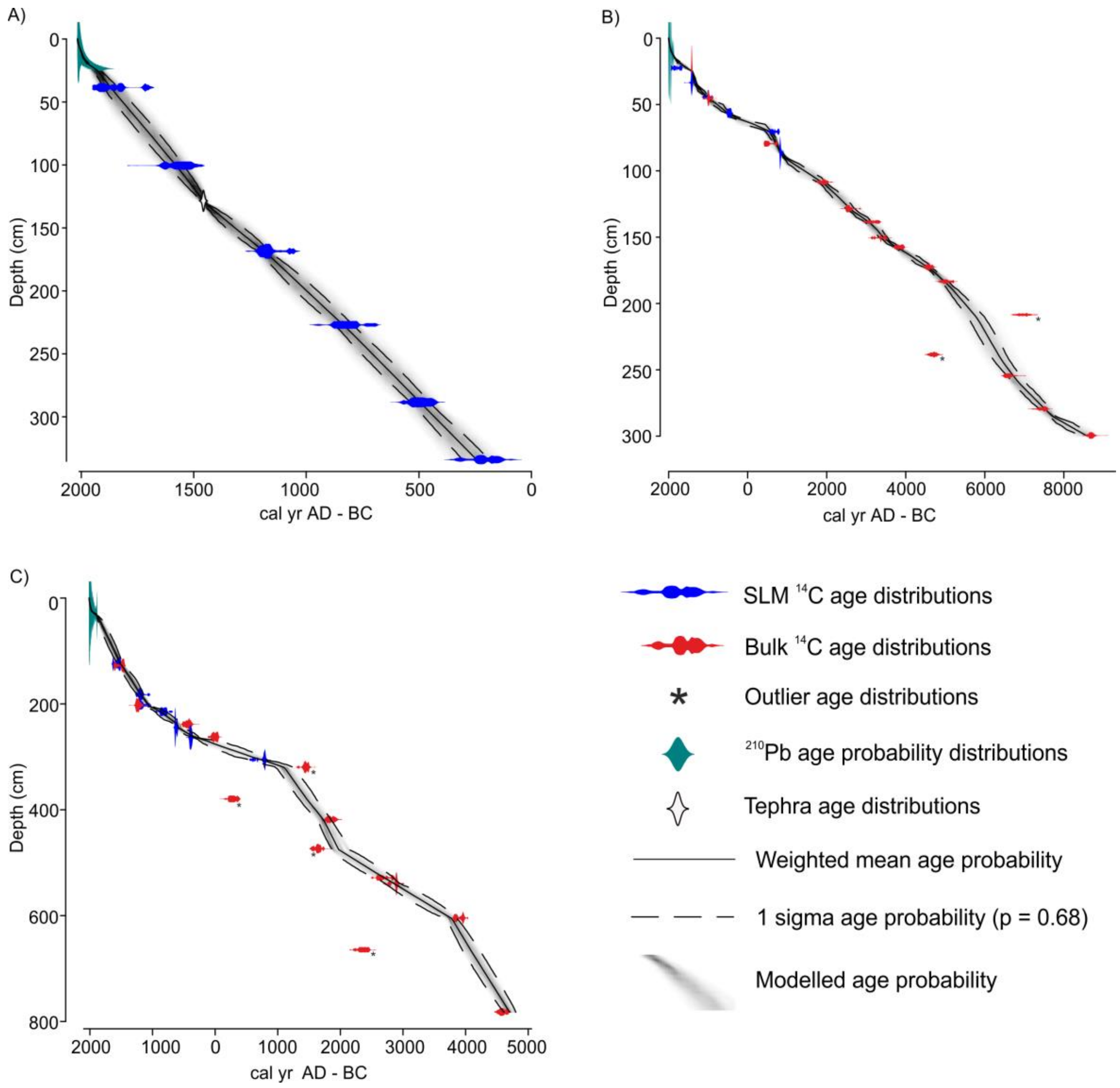

Fig. S2. Bayesian age models (built using BACON 2.2, SHCal13) for the lake sediment archives: A) Lake Emoatul (Efate Island, Vanuatu), B) Lake Lanoto'o ('Upolu Island, Samoa), and C) Lake Te Roto (Atiu Island, southern Cook Islands). Based on data in SI Tables 1-3.

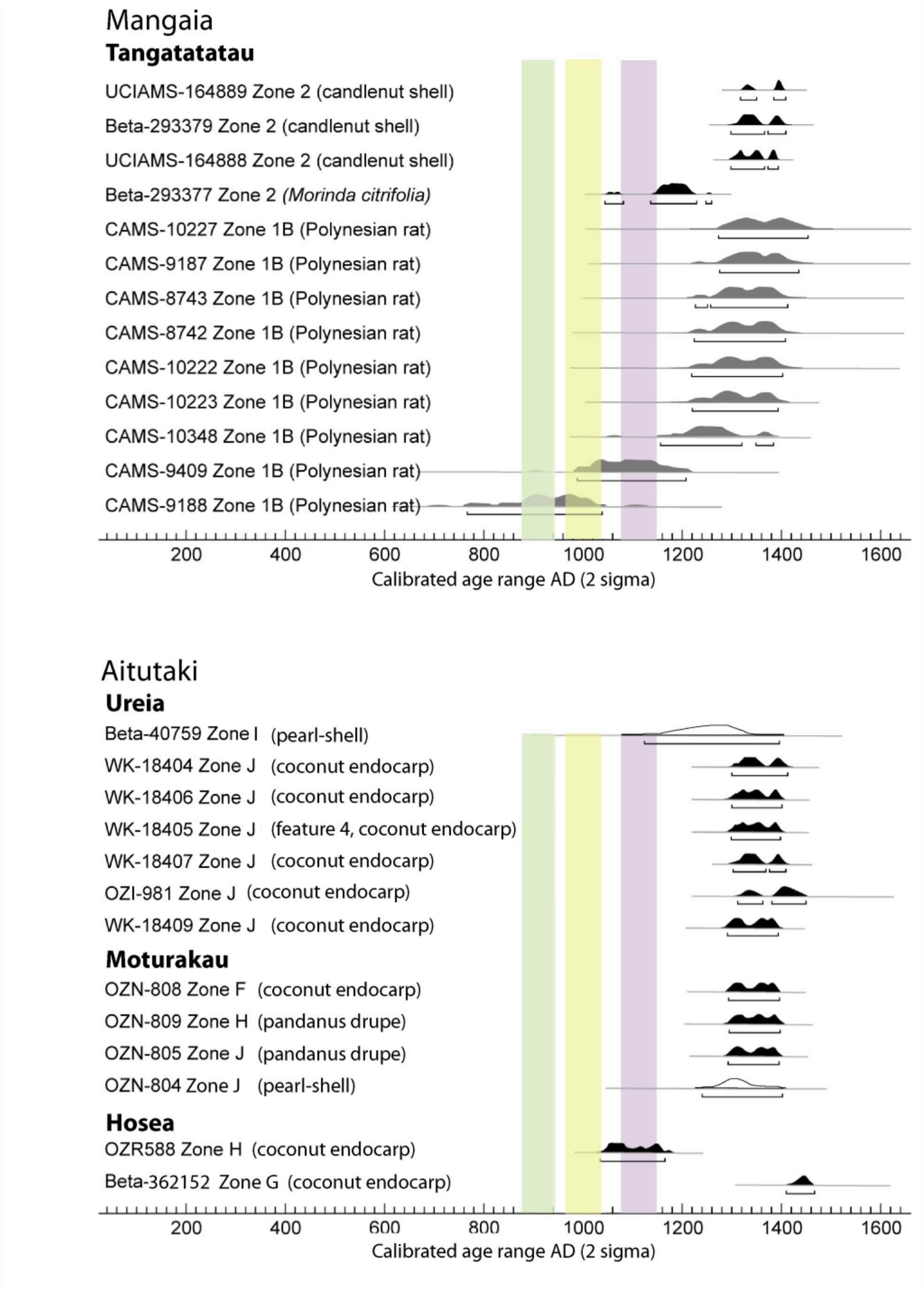

Fig. S3. – Early SCI radiocarbon dates on SLM plant material (black), rat bone (gray), and marine shell (white) from archaeological contexts on islands of Mangaia[12] and Aitutaki[40,14,15]. Overlap with the Lake Te Roto lake sequence is indicated by the colored bars that show Aitu core dates for discovery (green), colonization (yellow), and settlement (purple). The archaeological records, with one exception, post-date Atiu Island discovery as identified by the faecal sterol records of the present study (see main text for detailed discussion).

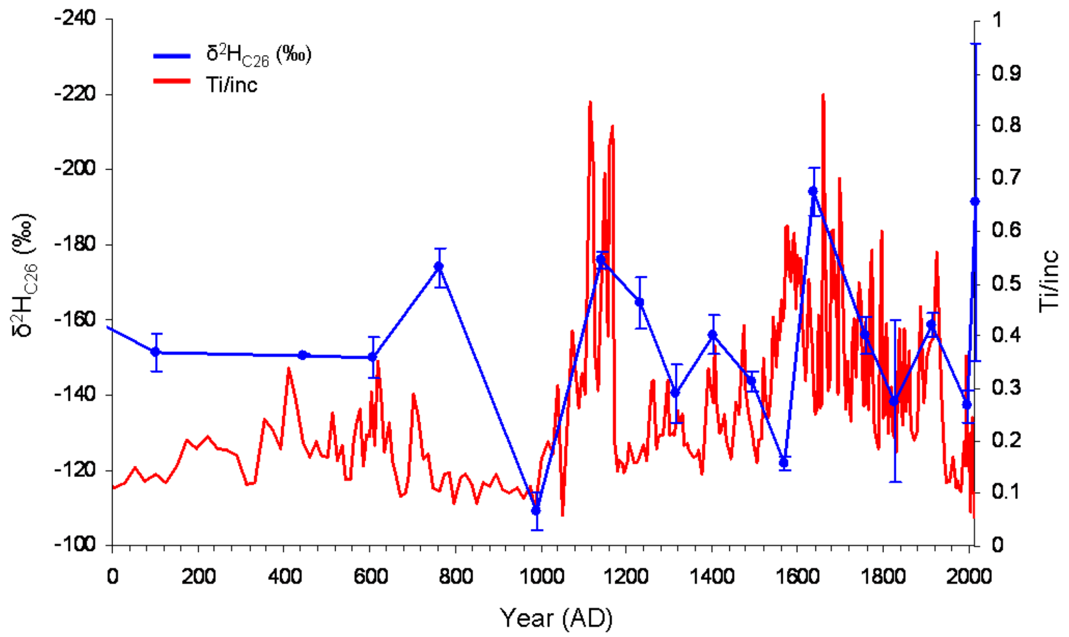

Fig. S4. Comparison between δ^2H_{C26} leaf wax (precipitation proxy) and Ti/inc (runoff proxy). δ^2H_{C26} axes inverted so drier conditions are less negative. Dry phase c. AD 850-950 corresponds with lower Ti/inc and least negative δ^2H_{C26} in the last 2,000 years. Peaks in Ti/Inc. and δ^2H_{C26} also correspond indicating wetter conditions. We conclude that, qualitatively, the Atiu biomarker record based on compound specific terrestrial plant leaf wax hydrogen reflects changes in precipitation amount.

Table S1. Lake Emoatul, Efate, Vanuatu. Sample ID's and details used in the BACON v2.2 age model.

CORE ID	LAB ID	Bacon 2.2 Model Age (cal BP)	Error	Depth (cm)	$\delta^{13}C_{VPDB}$(‰)	Sample Type
VAN_3474-1	GAU	-65	1	0.3	NA	surface
VAN_3508-1	GAU	-65	1	0.8	NA	210Pb
VAN_3537-1	GAU	-64	1	1.3	NA	210Pb
VAN_3537-2	GAU	-64	1	1.8	NA	210Pb
VAN_3508-2	GAU	-64	1	2.3	NA	210Pb
VAN_3537-3	GAU	-64	1	2.8	NA	210Pb
VAN_3537-4	GAU	-63	1	3.3	NA	210Pb
VAN_3537-5	GAU	-62	1	3.8	NA	210Pb
VAN_3474-2	GAU	-61	1	4.3	NA	210Pb
VAN_3537-6	GAU	-61	1	4.8	NA	210Pb
VAN_3537-7	GAU	-60	1	5.3	NA	210Pb
VAN_3537-8	GAU	-59	1	5.8	NA	210Pb
VAN_3508-3	GAU	-57	2	6.3	NA	210Pb
VAN_3537-9	GAU	-56	2	6.8	NA	210Pb
VAN_3537-10	GAU	-56	2	7.3	NA	210Pb
VAN_3537-11	GAU	-54	2	7.8	NA	210Pb
VAN_3474-3	GAU	-54	2	8.3	NA	210Pb
VAN_3537-12	GAU	-53	2	8.8	NA	210Pb
VAN_3537-13	GAU	-52	3	9.3	NA	210Pb
VAN_3537-14	GAU	-50	3	9.8	NA	210Pb
VAN_3508-4	GAU	-48	3	10.5	NA	210Pb
VAN_3537-15	GAU	-47	4	11.5	NA	210Pb
VAN_3474-4	GAU	-45	4	12.5	NA	210Pb
VAN_3537-16	GAU	-43	4	13.5	NA	210Pb
VAN_3508-5	GAU	-41	5	14.5	NA	210Pb
VAN_3537-17	GAU	-37	6	15.5	NA	210Pb
VAN_3474-5	GAU	-34	6	16.5	NA	210Pb
VAN_3537-18	GAU	-29	7	17.5	NA	210Pb
VAN_3508-6	GAU	-25	8	18.5	NA	210Pb
VAN_3537-19	GAU	-19	9	19.5	NA	210Pb
VAN_3474-6-L	GAU	-10	11	20.5	NA	210Pb
VAN_3537-20	GAU	0	13	21.5	NA	210Pb
VAN_3508-7	GAU	12	15	22.5	NA	210Pb
VAN_3537-21	GAU	35	20	23.5	NA	210Pb
**CORE ID**	**LAB ID**	**Conventional ^{14}C age (BP)**	**Error**	**Depth (cm)**	**$\delta^{13}C_{VPDB}$(‰)**	**Sample Type**
VANL1_90-91	SUERC-67469	349	37	100.5	-27.7	Monocot Leaf fragment
VANL1_28-29	D-AMS 026249	124	24	38.5	ND	Monocot Leaf fragment
VAN L2_128-129	CRONIN (pers comm.)	494	4	128.5	NA	Tephra (Kuwae)
VANL2_58.59	D-AMS 026251	923	26	168.5	ND	Monocot Leaf fragment
VANL3_15-16	SUERC-67470	1262	35	227	-27.9	Monocot Leaf fragment
VANL3_76-77	D-AMS 026252	1608	25	288.5	ND	Monocot Leaf fragment
VANL3A_56-57	SUERC-67471	1853	37	334	-26.6	Monocot Leaf fragment

Table S2. Lake Lanotoʻo, ʻUpolu, Samoa. Sample ID's and details used in the BACON v2.2 age model.

CORE ID	LAB ID	Bacon 2.2 Model Age (cal BP)	Error	Depth (cm)	$\delta^{13}C_{VPDB}$ (‰)	Sample Type
Surface	NA	-64	1	0	NA	surface
LAN14Pb1	GAU	-58	1	1	NA	210Pb
LAN14Pb2	GAU	-55	1	1.5	NA	210Pb
LAN14Pb3	GAU	-52	1	2	NA	210Pb
LAN14Pb4	GAU	-49	1	2.5	NA	210Pb
LAN14Pb5	GAU	-45	1	3	NA	210Pb
LAN14Pb6	GAU	-41	1	3.5	NA	210Pb
LAN14Pb7	GAU	-39	1	4	NA	210Pb
LAN14Pb8	GAU	-39	1	4.5	NA	210Pb
LAN14Pb9	GAU	-37	1	5	NA	210Pb
LAN14Pb10	GAU	-34	1	5.5	NA	210Pb
LAN14Pb11	GAU	-31	1	6	NA	210Pb
LAN14Pb12	GAU	-27	1	6.5	NA	210Pb
LAN14Pb13	GAU	-21	1	7	NA	210Pb
LAN14Pb14	GAU	-17	1	7.5	NA	210Pb
LAN14Pb15	GAU	-12	1	8.5	NA	210Pb
LAN14Pb16	GAU	-3	1	9	NA	210Pb
LAN14Pb17	GAU	2	1	9.5	NA	210Pb
LAN14Pb18	GAU	7	2	10	NA	210Pb
LAN14Pb19	GAU	25	5	11	NA	210Pb
LAN14Pb20	GAU	42	6	12	NA	210Pb
LAN14Pb21	GAU	65	5	13	NA	210Pb
**CORE ID**	**LAB ID**	**Conventional ^{14}C age (BP)**	**Error**	**Depth (cm)**	**$\delta^{13}C_{VPDB}$(‰)**	**Sample Type**
LAN14-U2 24-25cm	UCIAMS-179834	540	15	24.5	NA	Monocot Leaf fragment
LAN14-1-1 20-22cm	SUERC-63980	1096	35	45.5	-22.6	Bulk Sediment
LAN14-1-1 31-32cm	BETA-439599	1630	30	56.5	-26.8	Wood
LAN14-1-1 45-46cm	BETA-439600	2570	30	70.5	-26.7	Monocot Leaf fragment
LAN14-1-1 54-55cm	SUERC-63981	2453	37	79.5	-24.8	Bulk Sediment
LAN14-1-1 61-62cm	UCIAMS-179835	2700	20	86.5	NA	Monocot Leaf fragment
LAN14-2-1 30-31cm	SUERC-68884	3607	44	108.5	-25.9	Bulk Sediment
LAN14-2-1 60-61cm	SUERC-68885	4497	48	138.5	-27.3	Bulk Sediment
LAN14-1-2 13-14cm	SUERC-63982	4064	35	128.5	-24.2	Bulk Sediment
LAN14-1-2 35-36cm	SUERC-63983	4638	36	150.5	-25.6	Bulk Sediment
LAN14-1-2 43-44cm	SUERC-68886	5071	48	157.5	-25.2	Bulk Sediment
LAN14-1-2 57-58cm	SUERC-63984	5768	38	172.5	-24	Bulk Sediment
LAN14-1-2 69-70cm	SUERC-68887	6128	59	183.5	-23.4	Bulk Sediment
LAN14-2-2 11-12cm	SUERC-68891	8092	77	208.5	-23.6	Bulk Sediment
LAN14-1-3 17-18cm	SUERC-68892	5879	56	238.5	-24.5	Bulk Sediment
LAN14-1-3 33-34cm	SUERC-68893	7794	74	254.5	-24.2	Bulk Sediment
LAN14-1-3 68-59cm	SUERC-68894	8462	82	279.5	-25.7	Bulk Sediment
LAN14-1-3 77-78cm	SUERC-63985	9440	40	299.5	-22.8	Bulk Sediment

Table S3. Lake Te Roto, Atiu, southern Cook Islands. Sample ID's and details used in the BACON v2.2 age model.

CORE ID	LAB ID	Bacon 2.2 Model Age (cal BP)	Error	Depth (cm)	$\delta^{13}C_{VPDB}$(‰)	Notes
Surface	N/A	-64	1	0	NA	
TER-U2_2-3	GAU	-63	1	2.5	NA	210Pb
TER-U2_4-5	GAU	-62	1	4.5	NA	210Pb
TER-U2_5-6	GAU	-61	1	5.5	NA	210Pb
TER-U2_7-8	GAU	-60	1	7.5	NA	210Pb
TER-U2_8-9	GAU	-59	1	8.5	NA	210Pb
TER-U2_9-10	GAU	-57	0	9.5	NA	210Pb
TER-U2_10-11	GAU	-55	2	10.5	NA	210Pb
TER-U2_11-12	GAU	-54	2	11.5	NA	210Pb
TER-U2_12-13	GAU	-51	2	12.5	NA	210Pb
TER-U2_13-14	GAU	-49	2	13.5	NA	210Pb
TER-U2_14-15	GAU	-47	2	14.5	NA	210Pb
TER-U2_15-16	GAU	-44	2	15.5	NA	210Pb
TER-U2_16-17	GAU	-42	2	16.5	NA	210Pb
TER-U2_19-20	GAU	-39	2	19.5	NA	210Pb
TER-U2_20-21	GAU	-36	3	21.5	NA	210Pb
TER-U2_22-23	GAU	-31	3	22.5	NA	210Pb
TER-U2_23-24	GAU	-26	4	23.5	NA	210Pb
TER-U2_24-25	GAU	-22	5	24.5	NA	210Pb
TER-U2_25-26	GAU	-15	5	25.5	NA	210Pb
TER-U2_26-27	GAU	-8	7	26.5	NA	210Pb
TER-U2_27-28	GAU	2	6	27.5	NA	210Pb
TER-U2_28-29	GAU	11	8	28.5	NA	210Pb
TER-U2_29-30	GAU	37	11	29.5	NA	210Pb
TER-U2_32-33	GAU	57	4	32.5	NA	210Pb
**CORE ID**	**LAB ID**	**Conventional ^{14}C age (BP)**	**Error**	**Depth (cm)**	**$\delta^{13}C_{VPDB}$(‰)**	**Sample Type**
T1-2_15-16	UCIAMS-192876	335	15	124.5	ND	Monocot Leaf fragment
T1-2_18-19	SUERC-63973	427	37	127.5	-28.1	Bulk Sediment
T1-3 11.5	WH154722	895	40	182.5	-27.2	Monocot Leaf fragment
T1-3 31.5	WH154721	895	60	202.5	-28.5	Monocot Leaf fragment
T1-2_94-95	SUERC-68901	846	37	202.5	-25.5	Bulk Sediment
T1-3_42-44_45-48	SUERC-75681	1249	37	214.75	-28.0	Monocot Leaf fragment
T1-3_68-69	SUERC-68902	1664	35	238.5	-33.1	Bulk Sediment
T1-3_74-75	UCIAMS-192878	1465	20	244.5	ND	Monocot Leaf fragment
T1-3_91-92	SUERC-63974	2023	37	262.5	-33.4	Bulk Sediment
T1-3_92-93	UCIAMS-192879	1700	20	263.5	ND	Monocot Leaf fragment
T1-4_57-58	SUERC-75682	2629	37	305.5	-25.0	*Rhus taitensis* seed
T1-4_74-75	SUERC-68903	3211	37	319.5	-27.6	Bulk Sediment
T2-4_63-64	SUERC-68904	2259	37	379.5	-33.2	Bulk Sediment
T1-5_58-59	SUERC-68905	3568	38	418.5	-26.2	Bulk Sediment
T2-5_59-60	SUERC-68906	3390	37	473.5	-25.9	Bulk Sediment
T1-6_60-61	SUERC-68907	4140	35	528.5	-27.3	Bulk Sediment
T1-6_69-70	SUERC-63975	4304	36	539.5	-28.7	Bulk Sediment
T1-7_53-54	SUERC-68911	5167	37	604.5	-25.6	Bulk Sediment

T2-7_50-51	SUERC-68912	3918	37	664.5	-27	Bulk Sediment
T2-8_67-69	SUERC-63976	5780	37	782	-24.2	Bulk Sediment

Table S4. Normalised (z-score) values for hydroclimate proxies averaged over periods during the colonization of "gateway" islands (e.g., southern Cook Islands), and colonization into marginal East Polynesia. Data shown in Manuscript Figures 4a, 4b.

Island	Longitude	Latitude	Hydroclimate Proxy	AD 900-1150 z-score	AD 1150-1300 z-score	Source
Kiribati Islands	-157.394	1.841	Ca/Ti	1.335	0.218	Higley et al. (2018)[39]
Washington, Line Is	-160.373	4.679	$\delta^2H_{Dinosterol}$	-0.054	-0.057	Sachs et al. (2009)[41]
Palau	-134.442	7.307	$\delta^2H_{Dinosterol}$	0.998	-0.454	Sachs et al. (2018)[42]
Galapagos Is.	-89.480	-0.895	$\delta^2H_{Dinosterol}$	-1.296	0.597	Atwood & Sachs (2014)[43]
Rendova, Solomon Is.	-157.320	-8.691	$\delta^2H_{Dinosterol}$	-0.852	0.309	Maloney (2018)[32]
Uvea (Wallis)	-176.213	-13.321	$\delta^2H_{Dinosterol}$	-0.448	1.111	Maloney (2018)[32]
ʻUpolu, Samoa	-171.827	-13.910	$\delta^2H_{Dinosterol}$	-1.169	0.416	This study
Tahaʻa, Society Is.	-151.492	-16.672	LN(Ca/Ti)	-0.072	0.124	Toomey et al. (2016)[44]
Efate, Vanuatu	-168.414	-17.732	$\delta^2H_{Dinosterol}$	0.004	1.015	This study
Atiu, S. Cook Is.	-158.123	-20.009	$\delta^2H_{Leaf\ wax}$	-0.597	0.559	This study
Rimatara, Austral Is.	-152.812	-22.641	Magnetics (χLF)	-0.802	-0.604	Prebble & Wilmshurst (2009)[17]
Rapa Nui (Easter)	-109.289	-27.122	$\delta^{13}C$	-0.279	-0.358	Rull et al. (2016)[45]

SI References

1. W. Gosling *et al*., Human occupation and ecosystem change on Upolu (Samoa) during the Holocene, *Journal of Biogeography* https://doi.org/10.1111/jbi.13783 (2020).

2. I.W. Croudace, A. Rindby and R.G. Rothwell, "ITRAX: description and evaluation of a new multi-function X-ray core scanner" in New techniques in sediment core analysis, Eds. I.W. Croudace and R.G.Rothwell, (Geological Society Special Publication 267) pp 51-63. (2006).

3. A.G. Hogg, *et al*., ShCal13 Southern Hemisphere calibration, 0-50,000 cal yr BP. *Radiocarbon* **55**(4), 1889-1903 (2013).

4. M. Blaauw and J. Christen, A Flexible paleoclimate age-depth models using an autoregressive gamma process. *Bayesian Analysis* **6**, 457-474 (2011).

5. R. Duff, Notes on the prehistory of Atiu, Cook Islands. *Royal Society of New Zealand Bulletin* **8**, 41-49 (1971).

6. M.M. Trotter, Ed. *Prehistory of the Southern Cook Islands*. Canterbury Museum Bulletin No. 6. Canterbury, New Zealand (1974).

7. D.W. Steadman, Extinct and extirpated birds from Aitutaki and Atiu, Southern Cook Islands. *Pacific Science* **45**(4), 325-347 (1991).

8. H. Kurashina, R.A. Stephenson, Y. Sinoto, "*Sacred Stones of Polynesia: Mapping and Stablizing Ancient Polynesian Monuments in the Cook Islands"*. 1988 EARTHWATCH Report prepared for the Government of the Cook Islands (1987).

9. R. Walter, "*The Southern Cook Islands in Eastern Polynesian Prehistory".* PhD dissertation, University of Auckland, New Zealand, (1990).

10. R. Walter, "*Anai'o: The Archaeology of a Fourteenth Century Polynesian Community in the Southern Cook Islands"*. New Zealand Archaeological Association Monograph No. 22. New Zealand Archaeological Association, Auckland, (1998).

11. A.L. Clark, N. Tayles, H.R. Buckley and F. Neuman, Rima Rau Burial Cave, Atiu, Cook Islands. *Journal of Island and Coastal Archaeology* **11**(1), 68-88 (2016).

12. P.V. Kirch, Ed. "*Tangatatau Rockshelter: The Evolution of an Eastern Polynesian Socio-Ecosystem. Monumenta Archaeologica 40*" (UCLA Cotsen Institute of Archaeology Press) pp. 328 (2017).

13. E.M. Niespolo, W.D. Sharp and P.V. Kirch, ^{230}Th dating of coral abraders from stratified deposits at Tangatatau Rockshelter, Mangaia, Cook Islands: Implications for building precise chronologies in Polynesia. *Journal of Archaeological Science* **101**, 21-33 (2018).

14. M.S. Allen and A.E. Morrison, Modelling site formation dynamics: Geoarchaeological, chronometric and statistical approaches to a stratified rockshelter sequence, Polynesia. *Journal of Archaeological Science* **40**(12), 4560-4575 (2013).

15. M.S. Allen, A.E. Morrison, A.M. Lorrey, J-X. Zhao and G.E. Jacobsen, Timing, magnitude and effects of late Holocene sea level drawdown on island habitability, Aitutaki, Cook Islands. *Archaeology in Oceania* **51**(2), 108-121 (2016).

16. C. Bronk Ramsey, OxCal program, version 4.3. Available at https://c14.arch.ox.ac.uk/oxcal.html (2020).

17. M. Prebble and J. Wilmshurst, Detecting the initial impact of humans and introduced species on island environments in Remote Oceania using palaeoecology. *Biological Invasions* **11**(7), 1529–1556 (2009).

18. A.N. Rhodes, A method for the preparation and quantification of microscopic charcoal from terrestrial and lacustrine sediment cores. *The Holocene*, **8**(1), 113-117, (1998).

19. S. J. Stevenson and S. Haberle, Macro Charcoal Analysis: A modified technique used by the Department of Archaeology and Natural History. *Palaeoworks Technical Papers 5*, URL: http://palaeoworks.anu.edu.au/paltr05.pdf (2005).

20. K. Prost, J.J. Birk, E. Lehndorff, R. Gerlach and W. Amelung, Steroid Biomarkers Revisited – Improved Source Identification of Faecal Remains in Archaeological Soil Material. *PLoS ONE* **12**(1): e0164882.doi:10.1371/journal.pone.0164882 1-30 (2017).

21. A.J. White, *et al.*, An evaluation of fecal stanols as indicators of population change at Cahokia, Illinois. *Journal of Archaeological Science* **93**, 129-134 (2018).

22. E. Argiriadis, *et al*., Lake sediment faecal and biomass burning biomarkers provide direct evidence for prehistoric human-lit fires in New Zealand. *Scientific Reports* **8**, 1-9 (2018).

23. C.E.M. Lloyd, K. Michaelides, D.R. Chadwick, J.A. Dungait and R.P. Evershed, Tracing the flow-driven vertical transport of livestock-derived organic matter through soil using biomarkers. *Org. Geochem*. **43**, 56–66 (2012).

24. M. I. Bull, M. Lockheart, M. Elhmmali, D. Robertsand R. Evershed, The origin of faeces by means of biomarker detection. *Environment International* **27**(8), 647 – 654 (2002).

25. Kornilova and A. Rosell-Melé, Application of microwave-assisted extraction to the analysis of biomarker climate proxies in marine sediments. *Organic Geochemistry* **34**(11), 1517-1523 (2003).

26. A.E. Maloney, *et al*., Reconstructing precipitation in the tropical South Pacific from dinosterol 2H/1H ratios in lake sediment. *Geochimica et Cosmochimica Acta* **245**, 190-206 (2019).

27. J.P. Sachs, Hydrogen isotope signatures in the lipids of phytoplankton, In Treatise on Geochemistry, D.H Holland and K.K. Turekian Eds. (Elsevier Ltd, Oxford) pp. 79–94 (2014).

28. D. Sachse, *et al*., Molecular paleohydrology: interpreting the hydrogen-isotopic composition of lipid biomarkers from photosynthesising organisms. *Annual Review of Earth and Planetary Sciences* **40**, 221-249 (2012).

29. J.D. Hassall, "Static or Dynamic: Reconstructing Past Movement of the South Pacific Convergence Zone". PhD Thesis, University of Southampton, Department of Geography & Environmental Sciences UK. pp. 334 (2017).

30. D.B. Nelson and J.P. Sachs, Concurrent purification of sterols, triterpenols and alkenones from sediments for hydrogen isotope analysis using high performance liquid chromatography. *Org. Geochem*. **64**, 19–28 (2013).

31. A.L. Sessions, T.W. Burgoyne, and J.M. Hayes, Determination of the H_3 factor in hydrogen isotope ratio monitoring mass spectrometry. *Anal. Chem.* **73**, 200–207 (2001).

32. A.E. Maloney, "*Tropical South Pacific Paleohydrology from Hydrogen Isotopes in Algal Lipids*". Ph.D Dissertation, Oceanography, University of Washington pp.205 (2018).

33. D.B. Nelson and J.P. Sachs, The influence of salinity on D/H fractionation in dinosterol and brassicasterol from globally distributed saline and hypersaline lakes. *Geochim. Cosmochim.Acta* **133**, 325–339 (2014).

34. P.J. Polissar and W.J. D'Andrea, Uncertainty in paleohydrologic reconstructions from molecular δD values. *Geochim. Cosmochim. Acta* **129**, 146–156 (2014).

35. P.E. Sauer, T.I. Eglinton, J.M. Hayes, A. Schimmelmann and A.L. Sessions, Compound-specific D/H ratios of lipid biomarkers from sediments as a proxy for environmental and climatic conditions. *Geochimica et Cosmochimica Acta* **65**, 2113-222 (2001).

36. I.S. Castañeda and S.A. Schouten, Review of molecular organic proxies for examining modern and ancient lacustrine sediment, *Quaternary Science Reviews* **30**, 2851-2891 (2011).

37. G. Eglinton and R.J. Hamilton, Leaf epiticular waxes. *Science* **156**, 1322-1335 (1967).

38. P.J. Polissar, K.H. Freeman, D.B. Rowley, F.A. McInerney and B.S. Currie, Paleoaltimetry of the Tibetan Plateau from D/H ratios of lipid biomarkers. *Earth Planetary Science Letters* **287**, 64-76 (2009).

39. M.C. Higley, J.L. Conroy and S. Schmitt, Last Millennium Meridional Shifts in Hydroclimate in the Central Tropical Pacific. *Paleoceanography and Paleoclimatology* **33**, 354-366 (2018).

40. M.S. Allen and R. Wallace, New evidence from the East Polynesian gateway: Substantive and methodological results from Aitutaki, southern Cook Islands. *Radiocarbon* **49**(3), 1163-1179 (2007).

41. J.P. Sachs *et al.*, Southward movement of the Pacific intertropical convergence zone AD 1400-1850. Nature Geoscience **2**(7), 519-525 (2009).

42. J.P. Sachs *et al.*, Southward Shift of the Pacific ITCZ During the Holocene. *Paleoceanography and Paleoclimatology* **33**(12), 1383-1395 (2018).

43. A.R. Atwood and J.P. Sachs, Separating ITCZ- and ENSO-related rainfall changes in the Galápagos over the last 3 kyr using D/H ratios of multiple lipid biomarkers. *Earth and Planetary Science Letters* **404**, 408-419 (2014).

44. M.R. Toomey, J.P. Donnelly and J.E. Tierney, South Pacific hydrological and cyclone variability during the last 3000 years. *Paleoceanography* **31**, 491-504 (2016).

45. V. Rull, *et al*., CLAFS, a holistic climatic-ecological-anthropogenic hypothesis on Easter Island's deforestation and cultural change: Proposals and testing prospects. *Front. Ecol. Evol*. **6**, (32) doi: 10.3389/fevo.2018.00032 (2018).
